# Validation of a Questionnaire on the Post-COVID-19 Condition (Long COVID): A Cross-Sectional Study in Italy

**DOI:** 10.3390/idr17030069

**Published:** 2025-06-11

**Authors:** Angelo Cianciulli, Emanuela Santoro, Roberta Manente, Antonietta Pacifico, Gianni Comunale, Marika Finizio, Mario Capunzo, Francesco De Caro, Gianluigi Franci, Giuseppina Moccia, Giovanni Boccia

**Affiliations:** 1 Department of Medicine, Surgery and Dentistry ‘’Scuola Medica Salernitana”, University of Salerno, 84081 Salerno, Italy; ancianciulli@unisa.it (A.C.); apacifico@unisa.it (A.P.); g.comunale6@studenti.unisa.it (G.C.); m.finizio6@studenti.unisa.it (M.F.); mcapunzo@unisa.it (M.C.); fdecaro@unisa.it (F.D.C.); gfranci@unisa.it (G.F.); gmoccia@unisa.it (G.M.); gboccia@unisa.it (G.B.); 2 Clinical Pathology Unit, San Giovanni di Dio e Ruggi d’Aragona University Hospital, 84081 Salerno, Italy; manente392@gmail.com; 3 Integrated Care Department of Health Hygiene and Evaluative Medicine, San Giovanni di Dio e Ruggi d’Aragona University Hospital, 84131 Salerno, Italy; 4 Public Health Laboratory for the Analysis of Community Health Needs, Department of Medicine and Surgery, University of Salerno, Baronissi Campus, 84081 Baronissi, Italy; 5 Microbiology and Virology Unit, San Giovanni di Dio e Ruggi d’Aragona University Hospital, 84131 Salerno, Italy; 6 Hospital and Epidemiological Hygiene Unit, San Giovanni di Dio and Ruggi D’Aragona University Hospital 18 Hospital, 84131 Salerno, Italy

**Keywords:** long COVID, long convalescence, SARS-CoV-2 infection, muscle soreness, fatigue, loss of concentration, dyspnea, post-COVID-19 condition

## Abstract

Background/Objectives: Long COVID is a condition that was initially recognized by social support groups, and later by the scientific and medical communities. It affects COVID-19 survivors at various levels of severity, including young people, children and non-hospitalized people. Although the exact definition is unclear, the most common symptoms are fatigue and shortness of breath, which persist for months. Other symptoms include cognitive impairment, pain, palpitations, and gastrointestinal and heart problems. This study evaluated the reliability and validity of a questionnaire designed to examine the development and effects of long COVID. Methods: A questionnaire, composed of three sections, with a total of 24 items, was administered to subjects who had recovered from the COVID-19 disease in Italy. Data were collected from February to April 2025, and a statistical analysis was performed using R^®^ statistical software for Windows, version 4.3.3. Cronbach’s alpha was tested to check internal consistency. The questionnaire was completed voluntarily and anonymously by 250 individuals who had recovered from the SARS-CoV-2 infection. The questionnaire was self-administered and had open and structured questions. Results: The highest value of Cronbach’s alpha was found on 18 items (alpha = 0.97), which means that the questionnaire has satisfactory internal validity. Conclusions: This study highlights and confirms the continuity of symptoms manifested during the acute phase of the SARS-CoV-2 infection in the post-COVID-19 phase and the significant impact of these symptoms on daily life activities. Given its excellent reliability properties and high internal consistency, the instrument is recommended for future longitudinal studies and with large cohorts in order to carry out valid and replicable measurements of COVID-19 symptomatology.

## 1. Introduction

The post-COVID-19 condition, commonly known as long COVID, can affect anyone who has been exposed to the SARS-CoV-2 infection [1]. Strictly defining this condition proved challenging in the early stages of the pandemic. In the literature, the onset of long-term COVID-19 disorders appears under many names, including long COVID, post-acute COVID-19 syndrome (PACS), chronic COVID-19 syndrome, and long-haul COVID-19. In October 2021, the World Health Organization (WHO) proposed a term and clinical definition, namely, “post-COVID-19 condition” to unify the various existing definitions. Post-COVID-19 is, therefore, defined as a condition that occurs in individuals with a history of possible or confirmed SARS-CoV-2 infection, usually 3 months after onset, with persistent symptoms from the initial or de novo disease, variable and relapsing over time, which last at least 2 months and cannot be explained by an alternative diagnosis [2]. Furthermore, with the code U09.9 became part of the International Classification of Diseases, tenth revision (ICD-10) [3]. While the exact number of those living with the condition is uncertain, it is believed that more than 17 million people in the WHO European Region may have experienced it during the first two years of the pandemic (2020/21) [1]. Those who present sequelae following acute infection due to SARS-CoV-2 are commonly referred to as ‘long haulers’ or ‘long COVID patients’. Long COVID refers to the persistence of symptoms beyond the typical recovery period, often lasting several months after the acute infection has resolved [4].

Long COVID patients may report a wide range of symptoms affecting many different organs and body systems, especially at the systemic, respiratory, cardiac and neurological levels [5]. These symptoms can differ significantly from person to person and the severity can range from mild to disabling. Those commonly reported in studies include fatigue, muscle pain, shortness of breath, chest pain, cognitive dysfunction “brain fog” and post-exertional malaise [6]. A recent meta-analysis estimated that 80% of people infected with SARS-CoV-2 develop at least one long-term symptom [7]. Depending on the duration of symptoms, long COVID can be divided into two phases: acute post-COVID, in which symptoms extend beyond 3 weeks but last for less than 12 weeks, and chronic COVID, in which symptoms last for more than 12 weeks [8]. There is substantial evidence indicating that long COVID symptoms change over time. Initially, symptoms are often respiratory in nature, such as dyspnea and a persistent cough. However, these symptoms often evolve, with many patients developing neurological symptoms such as cognitive impairment and memory issues as the disease progresses. This progression from respiratory to neurological symptoms is well-documented in the literature [9]. However, based on the progression of the SARS-CoV-2 infection, it has been proposed to divide the post-acute manifestations into three categories: residual symptoms that persist after recovery from the acute infection; organ dysfunction that persists after initial recovery; and new symptoms or syndromes that develop after an initial asymptomatic or mild infection [10]. Finally, depending on the predominant residual symptoms, long COVID can be divided into different categories such as post-COVID-19 cardio-respiratory syndrome, post-COVID-19 fatigue syndrome and post-COVID-19 neuro-psychiatric syndrome. Categorizing symptoms based on the organ system involved will help identify the etiology [11]. The causes of long COVID are not yet well defined; there are probably multiple, potentially overlapping ones. Several mechanisms have been proposed to explain its etiopathogenesis, including the permanence of SARS-CoV-2 in some tissues. Ref. [12] has reported on the reactivation of neurotrophic pathogens under conditions of COVID-19 immune dysregulation, as well as microbiome alteration, coagulation problems, dysfunctional brainstem and vagus nerve signaling. Continuous immune cell activity and hyperactivation of T cells, especially CD8+ have also been reported [13]. Ref. [14] has reported that physical deconditioning and psychological problems such as post-traumatic stress also contribute to symptoms [15,16,17]. Several studies have identified some risk factors commonly associated with the development of long COVID such as age, sex, ethnicity, comorbidities, hospitalization and course of the COVID-19 disease [18,19,20]. Advanced age, female sex, hospitalization, initial dyspnea, chest pain, abnormal auscultatory findings (sounds coming from the heart, lungs, or other organs), duration of symptoms during the acute phase, and comorbidities, particularly asthma, were significantly associated with an increased risk of developing persistent symptoms [14]. Some studies have also focused on the impact of vaccination on the incidence of long COVID, reaching different conclusions. One study found no significant differences in the development of long COVID between vaccinated and unvaccinated individuals [21]. Other studies indicate that vaccines provide partial protection, with a reduced risk of long COVID ranging between 15% and 41% [22,23]. Koudi et al., for example, highlight how vaccination with two or more doses of BNT162b2 was associated with a reduced risk of developing the main post-acute symptoms of COVID-19, suggesting a protective role of the vaccine against the symptoms of long COVID [24]. Given the broad spectrum of cognitive and multi-system symptoms experienced by a large part of the population following the peak of the virus, it was necessary to examine the impact of these deficits on the daily lives of those affected. In a 2022 study conducted by Malik et al. on health-related quality of life assessed in all patients with long COVID measured via the EQ-5D-5L questionnaire, 58% of subjects reported poor quality of life following infection, with pain/discomfort and anxiety/depression reported by almost half of them, and a significantly more negative result was reported by patients who underwent hospitalization in intensive care during the acute infection [25]. In general, therefore, long haulers have a significantly worsened perception of their quality of life following the infection compared to subjects who contracted COVID-19 without reporting persistent symptoms [26]. Because long COVID occurs in various forms and with a wide range of clinical presentations, timely diagnosis is crucial to avoid negative consequences at various levels: individual, community, national and international [27]. Therefore, it is essential to identify patients with long COVID early and promptly initiate treatment using a multidisciplinary approach that includes assessment, symptomatic treatment, treatment of underlying problems, physiotherapy, occupational therapy and psychological support [8]. In this study, we hypothesized that a questionnaire specifically designed to assess the long-term symptoms of COVID-19 would provide valuable insights into the impact of long COVID on patients’ quality of life. The specific aim of this study is to validate the questionnaire as a reliable tool for assessing long COVID symptoms, measuring both their severity and impact on daily functioning. Through this validation process, we aim to ensure that the questionnaire can be utilized in clinical settings and research to monitor long COVID patients and inform potential therapeutic interventions.

## 2. Materials and Methods

The instrument underwent validation to test the internal validity and reliability of the items. Data were collected through a self-administered questionnaire. In the first phase, a pre-test was carried out on a sample of 30 subjects in order to identify any gaps and/or anomalous questions that could generate potential distortions of the research objective. The tool was then tested again on a non-probability sample of 250 subjects residing in Italy who had recovered from the SARS-CoV-2 infection. The questionnaire is preceded by a brief description explaining the details and purpose of the study. Each participant took part in the project voluntarily and anonymously, in order to guarantee maximum confidentiality on the data collected, and signed an informed consent where the methods and purposes of the study were explained in detail.

### 2.1. Study Design and Participant Demographics

The participants were selected using non-probability sampling and comprised 250 individuals who had recovered from SARS-CoV-2 infection. Participants were adults aged 18 or older, residing in Italy. Demographic information, such as age, sex, ethnicity, and comorbidities, were recorded to ensure a representative sample.

The inclusion criteria for the study were

- confirmed diagnosis of SARS-CoV-2
- evidence of long COVID symptoms (e.g., fatigue, dyspnea, cognitive impairment)
- informed consent and willingness to complete the questionnaire.

The exclusion criteria were

- individuals with severe underlying health conditions (e.g., cancer, severe neurological disorders)
- individuals unable to understand the questionnaire due to cognitive or language barriers.

### 2.2. Questionnaire Development and Structure

The items were generated based on a literature review and through consultation with medical and nursing staff who worked during the pandemic years in the COVID departments. The first complete version of the questionnaire was constructed ad hoc and consisted of 28 items divided into three sections. Further changes to the items were made after an initial administration. The new version of the questionnaire includes 24 questions and is structured in three sections that analyze sociodemographic information, the disease course and psychophysical condition after infection.

1. **Sociodemographic information**: The introductory section collects demographic data such as gender, age, nationality, and region of residence, the general conditions were identified with six questions plus a filter question to understand if the subject had ever had the SARS-CoV-2 infection and, therefore, if it was useful to continue the test.
2. **SARS-CoV-2 infection development and course**: In the second section concerning the development and course of the SARS-CoV-2 infection, ten questions were structured around the development and course of the COVID-19 disease, with particular interest in the degree of severity and duration of the symptoms manifested during the infection.
3. **Post-COVID-19 psychophysical condition**: In the third section, the post-COVID-19 psychophysical condition was framed with eight open and structured questions that identify the presence of specific signs and symptoms of long COVID and evaluate how much they impact on health, psychophysical well-being and daily life habits.

### 2.3. Likert Scale and Scoring

For the symptom severity analysis, a Likert scale (1–5) was used in each section to categorize the symptoms. The scales used were as follows:

- 1 (No symptoms), 2 (Mild symptoms), 3 (Moderate symptoms), 4 (Severe symptoms), and 5 (Critical symptoms) to measure symptom severity in both the acute and post-COVID phases.
- Additionally, for each symptom, participants were asked to rate their experience as Not at all, A little, Moderately, Quite, or Extremely using a Likert scale (1–5).

### 2.4. Data Collection and Analysis

Data were collected from February to April 2025, entered into a database, and processed using the statistical software R^®^ (R Foundation for Statistical Computing, Vienna, Austria) for Windows, version 4.3.3. Descriptive analyzes were performed using frequencies, percentages, and a frequency table for categorical variables. The reliability of the questionnaire was assessed by calculating Cronbach’s alpha [20], a measure of internal consistency. This parameter can be interpreted as an average of the correlation coefficients calculated for each possible subdivision of the items into two groups of equal size. The reliability of the evaluation of a scale consists in the estimate of how much the variation in score may be real or effective, and not due to chance or random errors. The degree of reliability estimated by Cronbach’s alpha is expressed as a proportion; for example, a degree of reliability equal to 0.70 means that the measured variance can be considered 70% reliable [28].

### 2.5. Ethical Considerations

The study was conducted in compliance with the ethical standards set by the Helsinki Declaration. Ethical approval was granted by the Campania 2 Ethics Committee (protocol no. 2025/3339), which ensures that all procedures were carried out in a manner that prioritized the safety, privacy, and autonomy of the participants.

## 3. Results

### 3.1. Validation of the Questionnaire

The Cronbach’s alpha value for two sections of the questionnaire—development and course of the SARS-CoV-2 infection and post-COVID-19 psychophysical condition—was 0.97, which indicates excellent internal consistency (Table 1). However, such a high alpha may suggest redundancy among items, and further investigation into the item structure and possible refinement of the questionnaire is recommended to ensure diversity of content. The minimum acceptable level for internal consistency is typically between 0.60 and 0.80 [29].

#### 3.1.1. Sociodemographic Characteristics of the Sample

The sample consisted of 250 individuals who had recovered from COVID-19. The majority of participants were female (63.6%), while 36.4% were male, as shown in Table 2. The age distribution revealed that the highest percentage of participants (56%) were aged 30 years or younger. A recent pathological history was conducted, with 95.6% of the participants reporting no pre-existing health conditions prior to COVID-19 infection. The participants were also asked to assess the severity of their COVID-19 illness on a scale from 1 (mild) to 5 (severe). The results indicated that 11.6% of respondents rated their illness severity as 1, 40% as 2, 40% as 3, 6.8% as 4, and 1.6% as 5. 

#### 3.1.2. COVID-19 Symptoms

COVID-19 can cause a wide spectrum of clinical manifestations, which can vary in severity. Among the most frequently reported symptoms during the acute phase of the disease, as shown in Table 3, the mean values and standard deviations (SD) were as follows: persistent fatigue/asthenia 2.60 (SD ± 1.17), excessive tiredness 2.76 (SD ± 1.31), muscle weakness 2.77 (SD ± 1.28), headache 2.35 (SD ± 1.36), nasal congestion or runny nose 2.52 (SD ± 1.35), muscle and joint pain 2.79 (SD ± 1.31), mood disorders 2.17 (SD ± 1.24), intermittent fever 2.19 (SD ± 1.20), smell disturbances 2.27 (SD ± 1.44), tachycardia 1.74 (SD ± 1.21), difficulty concentrating 2.09 (SD ± 1.25), swallowing and taste dysfunction 2.18 (SD ± 1.38), long cold 2.15 (SD ± 1.27) and a persistent dry cough 2.28 (SD ± 1.31). These values indicate that fatigue, muscle weakness, and pain were the most commonly reported symptoms, with a moderate-to-high degree of severity. The term “long cold” refers to prolonged mild respiratory symptoms such as cough, nasal congestion, and runny nose that persists after the acute phase of the infection has resolved. This condition, although not universally recognized in clinical guidelines, is commonly reported by patients as part of long COVID recovery and is characterized by lingering but less severe symptoms. We acknowledge that this term is not standardized, but it is used here to describe ongoing, mild symptoms observed post-COVID infection. In line with findings from the Post-COVID Functional Status (PCFS) scale, our study also identified persistent symptoms such as fatigue, dyspnea, and muscle weakness, which significantly impacted participants’ quality of life and functional capacity. These symptoms, as reported by PCFS, persist long after the acute phase of the infection, reinforcing the importance of targeted interventions for long COVID patients [30,31,32,33].

#### 3.1.3. Post-COVID-19 Condition

Following recovery and viral clearance, some symptoms persist and continue to impact individuals’ daily lives. These lingering symptoms represent a continuation of the disease and can affect both physical and psychological well-being. As shown in Table 4, the mean values and standard deviations (SD) for the most frequent symptoms observed in the post-viral phase were as follows: persistent fatigue/asthenia, 2.20 (SD ± 1.24), excessive tiredness 2.36 (SD ± 1.27), muscle weakness 2.25 (SD ± 1.29), muscle and joint pain 2.25 (SD ± 1.25) and headache 1.94 (SD ± 1.26). In comparison to the acute phase, lower values were recorded for other symptoms. Despite this reduction, these symptoms still significantly impacted daily activities, leading to limitations in functional tasks and a variable degree of psychophysical well-being. A complete list of absolute frequencies for all reported symptoms during and after SARS-CoV-2 infection is available in the Supplementary Materials (Tables S1–S5).

## 4. Discussion

Several studies have attempted to measure a range of symptoms that occur as a consequence of COVID-19 and, at the same time, to detect their burden on the quality of life of affected individuals [34,35,36,37,38,39]. Because the clinical manifestations of long COVID are extremely variable, there is no specific framework that defines their characteristics; in fact, they are generally divided into general and organ-specific manifestations [40]

In our study, it should be considered that the lowest possible severity could be classified as level 1. This means that a rating of 1 could mean no symptoms or mild symptoms. The most frequently manifested general symptoms after SARS-CoV-2 infection were persistent fatigue/asthenia, excessive tiredness, muscle weakness, and muscle pain. Among the organ-specific manifestations, the most commonly reported symptoms were dyspnea at rest and following light exertion, headache, tachycardia, mood disorders, sleep disorders, difficulty concentrating, and a feeling of chest tightness. These symptoms were then compared with those observed during the acute phase of the disease, highlighting their continuity. The results of our study confirm that long COVID symptoms tend to persist over time, with a lasting impact on the daily lives of individuals.

Long COVID symptoms impact individuals on a daily basis, limiting their ability to return to work, participate in social activities, or take care of themselves [41]. In a multicenter study of outpatients with COVID-19, who were contacted between 6 and 11 months after they tested positive, the number of symptoms reported was associated with lower overall health status, lower quality of life, and greater psychological stress [42]. These results align with our study, which shows that the majority of the sample reported that symptoms had significantly influenced their daily lives, particularly in activities such as work, study, recreational activities, and health management.

### 4.1. Implications of the Results

The results of this study contribute to the growing body of literature on long COVID, confirming the persistence of symptoms and their significant impact on quality of life. The symptoms reported by participants, such as persistent fatigue and dyspnea, have also been widely described in other studies, emphasizing the importance of developing diagnostic tools and therapeutic interventions specifically targeting long COVID. However, despite the effectiveness of the questionnaire in measuring the symptoms and impact of long COVID, further studies are needed to confirm the generalizability of the results to other populations and to further improve the questionnaire’s validity.

The questionnaire developed in this study could be a useful tool for monitoring long COVID patients and guiding the implementation of therapeutic interventions. Future research could focus on validating the questionnaire in different populations and in longitudinal studies to better understand how symptoms evolve over time and how the effectiveness of interventions may vary depending on the duration of long COVID.

### 4.2. Limitations of the Study

There are some limitations to consider. The sample used was selected through non-probability sampling, which could introduce selection bias. While this approach was practical due to time constraints, the results may not be fully generalizable to all individuals suffering from long COVID. Additionally, the study was conducted in Italy, which limits the geographical and cultural diversity of the sample. Cultural and regional differences may affect the experience and manifestation of long COVID symptoms, suggesting that multi-center and international studies could provide a more comprehensive understanding of the phenomenon.

Timing of the survey: data were collected between February and April 2025, several months after participants had recovered from the acute phase of COVID-19. This allowed us to focus on post-viral symptoms, but the exact timing between infection and survey participation may vary across individuals. Further clarification of this timing may be important for future studies to understand the relationship between symptom persistence and time since recovery.

Another limitation concerns the cross-sectional design of the study, which provides only a snapshot of symptoms at a specific point in time. While this approach is useful, it does not allow for the observation of symptom evolution and a better understanding of why some symptoms are resolved over time, while others persist or worsen. Therefore, longitudinal studies should be conducted to observe how symptoms evolve over time and to identify potential risk factors for the persistence of long COVID.

Hospitalization status: Only one participant in our study had been hospitalized during the acute phase of COVID-19. This limited number of hospitalizations may not fully represent the impact of severe cases on long COVID symptoms, as hospitalized patients may have different symptom profiles compared to those who were not hospitalized.

It is also important to note that the sample in this study was skewed toward individuals under 30 years old and predominantly from the Campania region. This limits the generalizability of the results, as younger individuals and those from this region may have different experiences of long COVID compared to other demographics. Future studies should aim for more diverse and representative samples to ensure broader applicability of the results.

### 4.3. Directions for Future Research

Future studies should focus on longitudinal research to better understand how long COVID symptoms change over time and identify potential interventions that can alleviate the burden of these symptoms. Psychosocial factors, such as mental health challenges and social support, may also play an important role in the experience of long COVID and should be examined more closely in future research. Studies could explore how psychological interventions, rehabilitation programs, or pharmacological treatments may help mitigate the long-term effects of the disease.

Additionally, multi-center and multi-country studies should be undertaken to capture a more diverse range of long COVID experiences. This would help enhance the generalizability of findings, as factors like ethnicity, region, and socioeconomic status may influence the prevalence and impact of long COVID symptoms.

Finally, further investigation into the mechanisms underlying long COVID is needed [43,44]. Research could focus on immune dysregulation, neurological involvement, microbiome changes, and autoimmunity to uncover potential targets for therapeutic interventions.

## 5. Conclusions

Multidisciplinary follow-up and adequate rehabilitation represent two aspects of extreme importance in the recognition and management of subjects affected by long COVID. This study demonstrates that the questionnaire, used as a tool to recognize the symptomatology of long COVID and evaluate its potential individual and social effect, has excellent reliability properties. In terms of internal consistency and validity, it also seems to have good performance, which should be taken into consideration in future studies. The results are not generalizable due to insufficient scientific knowledge of the symptoms of long COVID which, being a new emergency condition, cannot benefit from previous and consolidated evidence; however, the main objective was the validation of the questionnaire. This study is only the starting point of further studies that aim to deepen knowledge relating to the symptoms of long COVID and its short- and long-term effects on the psychophysical state of each individual.

## Figures and Tables

**Table 1:** Cronbach’s alpha reliability.

Section	Alpha
2	0.94
3	0.96
2 + 3	0.97

**Table 2:** Demographic, professional and personal characteristics of the sample.

Total Participants	*n*	%
	250	100.00
**Gender**	*n*	%
Men	91	36.40
Women	159	63.60
**Age**	*n*	%
30+	110	44.00
<=30	140	56.00
**Region of residence**	*n*	%
Basilicata	2	0.80
Calabria	2	0.80
Campania	208	83.20
Emilia Romagna	6	2.40
Lazio	12	4.80
Lombardia	9	3.60
Molise	2	0.80
Piemonte	2	0.80
Sicilia	5	2.00
Umbria	1	0.40
Veneto	1	0.40
** Occupational Status and Lifestyle**		
** 1. What is your occupational status?**		
Status	*n*	%
Homemaker	1	0.40
Unemployed or job seeking	27	10.80
Employed	148	59.20
Retired	4	1.60
Withdrawn from work	3	1.20
Student	67	26.80
** 2. Have you ever smoked?**		
Response	*n*	%
I smoke regularly	77	30.80
I smoke occasionally	19	7.60
I have never smoked	114	45.60
Yes, I smoked in the past	25	10.00
Yes, I smoked in the past	15	6.00
** Coronavirus and Health**		
** 2. Coronavirus disease can have a different course from one subject to another. Some may not present any symptoms (be asymptomatic); others, instead, present symptoms from mild to severe. Choose the most appropriate level to define your course**		
Level	*n*	%
1.00	29	11.60
2.00	100	40.00
3.00	100	40.00
4.00	17	6.80
5.00	4	1.60
** 3. Do you have pre-existing pathologies prior to SARS-CoV-2 infection?**		
Response	*n*	%
No	239	95.60
Yes	11	4.40
** 4. If yes, indicate which ones**		
Pathology	*n*	%
Asthma	2	18.18
Bronchial asthma	1	9.09
Hypertension	5	45.45
Hypothyroidism, sinus tachycardia, hyperprolactinemia	1	9.09
Neurological to be investigated	1	9.09
Respiratory, cardiological, weight problems	1	9.09
** 5. Do you take medications?**		
Response	*n*	%
No	236	94.40
Yes	14	5.60
** 6. If yes, which one(s)**		
Medication	*n*	%
Antihypertensives	4	28.57
Antihypertensives, levothyroxine	1	7.14
Beta-blocker	1	7.14
Cardicor, eutirox, dostinex	1	7.14
Cyclosporine	1	7.14
Deltacortene, Tachipirina 1000	1	7.14
Glialia	1	7.14
Insulin	1	7.14
Contraceptive pill	1	7.14
Pradaxa, Olprezide	1	7.14
Ventolin A/B	1	7.14

**Table 3:** Symptoms reported during SARS-CoV-2 illness.

Symptom	1	2	3	4	5	Mean	Median	sd
Persistent fatigue/asthenia	21.00	27.00	29.00	17.00	6.00	2.60	3.00	1.17
Excessive tiredness	24.00	18.00	26.00	22.00	10.00	2.76	3.00	1.31
Intermittent fever	38.00	24.00	24.00	8.00	6.00	2.19	2.00	1.20
Muscle weakness	22.00	21.00	25.00	23.00	9.00	2.77	3.00	1.28
Muscle and joint pain	23.00	20.00	20.00	29.00	8.00	2.79	3.00	1.31
Abdominal pain	60.00	17.00	14.00	8.00	0.00	1.70	1.00	1.00
Convulsions	90.00	8.00	2.00	0.00	0.00	1.13	1.00	0.42
Nausea and vomiting	78.00	12.00	6.00	2.00	2.00	1.38	1.00	0.83
Reduced or loss of appetite	44.00	25.00	14.00	12.00	5.00	2.10	2.00	1.23
Gastroesophageal reflux	72.00	12.00	9.00	4.00	2.00	1.51	1.00	0.97
Dyspnea during light efforts	52.00	23.00	15.00	5.00	5.00	1.88	1.00	1.14
Dyspnea at rest	70.00	15.00	8.00	3.00	4.00	1.56	1.00	1.03
Persistent dry cough	40.00	20.00	20.00	13.00	7.00	2.28	2.00	1.31
Sense of oppression	60.00	13.00	16.00	8.00	4.00	1.82	1.00	1.16
Chest pain	62.00	18.00	12.00	5.00	3.00	1.68	1.00	1.05
Tachycardia	66.00	10.00	12.00	5.00	6.00	1.74	1.00	1.21
Headache	40.00	18.00	19.00	15.00	9.00	2.35	2.00	1.36
Concentration difficulties	47.00	18.00	19.00	11.00	5.00	2.09	2.00	1.25
Memory problems	66.00	13.00	11.00	5.00	6.00	1.72	1.00	1.18
Nasal congestion or runny nose	30.00	25.00	18.00	16.00	11.00	2.52	2.00	1.35
Smell disorders (hyposmia or parosmia)	46.00	18.00	13.00	12.00	12.00	2.27	2.00	1.44
Swallowing or taste dysfunctions	48.00	15.00	18.00	8.00	10.00	2.18	2.00	1.38
Tinnitus, ear pain, dysphonia and sore throat	50.00	19.00	18.00	10.00	3.00	1.98	1.50	1.17
Skin rash	86.00	8.00	4.00	1.00	0.00	1.22	1.00	0.62
Sleep disorders	64.00	14.00	10.00	10.00	2.00	1.72	1.00	1.12
Mood disorders (general malaise)	42.00	22.00	20.00	11.00	6.00	2.17	2.00	1.24
Long cold	44.00	20.00	19.00	10.00	7.00	2.15	2.00	1.27
Depression or anxiety	59.00	17.00	10.00	8.00	6.00	1.85	1.00	1.23
Hair loss	76.00	10.00	6.00	5.00	3.00	1.49	1.00	1.00

**Table 4:** Symptoms reported after SARS-CoV-2 negative test.

Symptom	1	2	3	4	5	Mean	Median	sd
Persistent fatigue/asthenia	40.00	22.00	23.00	8.00	7.00	2.20	2.00	1.24
Excessive tiredness	33.00	25.00	22.00	11.00	8.00	2.36	2.00	1.27
Intermittent fever	72.00	12.00	12.00	4.00	0.00	1.49	1.00	0.87
Muscle weakness	40.00	20.00	20.00	12.00	7.00	2.25	2.00	1.29
Muscle and joint pain	38.00	25.00	19.00	13.00	6.00	2.25	2.00	1.25
Abdominal pain	74.00	14.00	9.00	2.00	1.00	1.42	1.00	0.81
Convulsions	95.00	3.00	1.00	1.00	0.00	1.07	1.00	0.36
Nausea and vomiting	83.00	10.00	6.00	2.00	0.00	1.27	1.00	0.68
Reduced or loss of appetite	67.00	16.00	11.00	3.00	3.00	1.58	1.00	0.98
Gastroesophageal reflux	72.00	15.00	8.00	2.00	2.00	1.47	1.00	0.90
Dyspnea during light efforts	58.00	25.00	11.00	3.00	3.00	1.68	1.00	0.99
Dyspnea at rest	79.00	9.00	6.00	4.00	3.00	1.43	1.00	0.96
Persistent dry cough	61.00	16.00	12.00	6.00	4.00	1.75	1.00	1.12
Sense of oppression	70.00	12.00	10.00	4.00	4.00	1.60	1.00	1.09
Chest pain	72.00	15.00	7.00	3.00	3.00	1.49	1.00	0.96
Tachycardia	74.00	11.00	8.00	4.00	4.00	1.52	1.00	1.03
Headache	56.00	14.00	16.00	8.00	6.00	1.94	1.00	1.26
Concentration difficulties	59.00	15.00	14.00	7.00	5.00	1.84	1.00	1.20
Memory problems	66.00	12.00	13.00	5.00	3.00	1.67	1.00	1.09
Nasal congestion or runny nose	58.00	12.00	18.00	6.00	5.00	1.87	1.00	1.21
Smell disorders (hyposmia or parosmia)	68.00	12.00	10.00	5.00	6.00	1.70	1.00	1.19
Swallowing or taste dysfunctions	66.00	16.00	6.00	6.00	5.00	1.68	1.00	1.16
Tinnitus, ear pain, dysphonia and sore throat	67.00	15.00	12.00	3.00	3.00	1.59	1.00	1.00
Skin rash	86.00	7.00	5.00	2.00	0.00	1.23	1.00	0.65
Sleep disorders	68.00	13.00	9.00	5.00	5.00	1.65	1.00	1.13
Mood disorders (general malaise)	56.00	16.00	16.00	6.00	6.00	1.89	1.00	1.21
Long cold	60.00	15.00	15.00	6.00	4.00	1.79	1.00	1.15
Depression or anxiety	64.00	16.00	10.00	5.00	5.00	1.70	1.00	1.13
Hair loss	76.00	12.00	5.00	4.00	3.00	1.45	1.00	0.97

## Data Availability

Data are contained within the article.

## Supplementary Materials

The following supporting information can be downloaded at: https://www.mdpi.com/article/10.3390/idr17030069/s1, Table S1: Table with absolute frequencies for symptoms; Table S2: Absolute frequencies of persistent symptoms after negative test; Table S3: How much have the declared symptoms affected your daily life? (complete absolute frequencies); Table S4: Have you experienced difficulties in resuming or performing the following activities? (complete absolute frequencies); Table S5: Today, do you believe that your life has changed following Covid-19 disease? (complete absolute frequencies).
