# Supplementary material for: Validation of a Questionnaire on the Post-COVID-19 Condition (Long COVID): A Cross-Sectional Study in Italy
Source: Infect Dis Rep. 2025 Jun 11;17(3):69. doi: 10.3390/idr17030069 (PMC12192970; doi:10.3390/idr17030069)
Supplement: Supplementary file 1 [file idr-17-00069-s001.zip › idr-3615314-supplementary-done.pdf]

infectious disease reports

MDPI

Table S1. with absolute frequencies for symptoms.

Symptom	1	2	3	4	5	mean	median	sd
Persistent fatigue/asthenia	53.00	67.00	73.00	42.00	15.00	2.60	3.00	1.17
Excessive tiredness	60.00	46.00	64.00	55.00	25.00	2.76	3.00	1.31
Intermittent fever	96.00	60.00	59.00	21.00	14.00	2.19	2.00	1.20
Muscle weakness	54.00	53.00	62.00	58.00	23.00	2.77	3.00	1.28
Muscle and joint pain	58.00	50.00	49.00	72.00	21.00	2.79	3.00	1.31
Abdominal pain	151.00	43.00	36.00	19.00	1.00	1.70	1.00	1.00
Convulsions	224.00	20.00	5.00	1.00	0.00	1.13	1.00	0.42
Nausea and vomiting	194.00	31.00	15.00	6.00	4.00	1.38	1.00	0.83
Reduced or loss of appetite	109.00	62.00	36.00	31.00	12.00	2.10	2.00	1.23
Gastroesophageal reflux	181.00	31.00	23.00	9.00	6.00	1.51	1.00	0.97
Dyspnea during light efforts	129.00	58.00	38.00	13.00	12.00	1.88	1.00	1.14
Dyspnea at rest	175.00	37.00	21.00	7.00	10.00	1.56	1.00	1.03
Persistent dry cough	100.00	49.00	50.00	33.00	18.00	2.28	2.00	1.31
Sense of oppression	150.00	33.00	39.00	19.00	9.00	1.82	1.00	1.16
Chest pain	156.00	45.00	29.00	13.00	7.00	1.68	1.00	1.05
Tachycardia	166.00	26.00	31.00	12.00	15.00	1.74	1.00	1.21
Headache	100.00	44.00	47.00	37.00	22.00	2.35	2.00	1.36
Concentration difficulties	117.00	46.00	47.00	27.00	13.00	2.09	2.00	1.25
Memory problems	164.00	32.00	28.00	12.00	14.00	1.72	1.00	1.18
Nasal congestion or runny nose	76.00	62.00	46.00	39.00	27.00	2.52	2.00	1.35
Smell disorders (hyposmia or parosmia)	114.00	44.00	33.00	29.00	30.00	2.27	2.00	1.44
Swallowing or taste dysfunctions	120.00	38.00	45.00	21.00	26.00	2.18	2.00	1.38
Tinnitus, ear pain, dysphonia and sore throat	125.00	47.00	45.00	25.00	8.00	1.98	1.50	1.17
Skin rash	214.00	21.00	11.00	3.00	1.00	1.22	1.00	0.62
Sleep disorders	161.00	35.00	24.00	24.00	6.00	1.72	1.00	1.12
Mood disorders (general malaise)	105.00	54.00	49.00	28.00	14.00	2.17	2.00	1.24
Long cold	110.00	51.00	48.00	24.00	17.00	2.15	2.00	1.27
Depression or anxiety	147.00	43.00	26.00	19.00	15.00	1.85	1.00	1.23
Hair loss	189.00	26.00	16.00	12.00	7.00	1.49	1.00	1.00

Table S2. Absolute frequencies of persistent symptoms after negative test.

Symptom	1	2	3	4	5	mean	median	sd
Persistent fatigue/asthenia	99.00	56.00	58.00	20.00	17.00	2.20	2.00	1.24
Excessive tiredness	83.00	63.00	56.00	28.00	20.00	2.36	2.00	1.27
Intermittent fever	179.00	31.00	30.00	9.00	1.00	1.49	1.00	0.87
Muscle weakness	101.00	51.00	50.00	31.00	17.00	2.25	2.00	1.29
Muscle and joint pain	94.00	62.00	47.00	32.00	15.00	2.25	2.00	1.25
Abdominal pain	186.00	34.00	22.00	6.00	2.00	1.42	1.00	0.81

Convulsions	238.00	8.00	2.00	2.00	0.00	1.07	1.00	0.36
Nausea and vomiting	207.00	24.00	14.00	4.00	1.00	1.27	1.00	0.68
Reduced or loss of appetite	168.00	41.00	27.00	7.00	7.00	1.58	1.00	0.98
Gastroesophageal reflux	181.00	37.00	21.00	6.00	5.00	1.47	1.00	0.90
Dyspnea during light efforts	145.00	62.00	28.00	8.00	7.00	1.68	1.00	0.99
Dyspnea at rest	197.00	22.00	15.00	9.00	7.00	1.43	1.00	0.96
Persistent dry cough	153.00	41.00	31.00	16.00	9.00	1.75	1.00	1.12
Sense of oppression	176.00	29.00	25.00	9.00	11.00	1.60	1.00	1.09
Chest pain	181.00	37.00	17.00	8.00	7.00	1.49	1.00	0.96
Tachycardia	184.00	28.00	20.00	9.00	9.00	1.52	1.00	1.03
Headache	140.00	36.00	39.00	20.00	15.00	1.94	1.00	1.26
Concentration difficulties	147.00	37.00	36.00	18.00	12.00	1.84	1.00	1.20
Memory problems	165.00	31.00	33.00	13.00	8.00	1.67	1.00	1.09
Nasal congestion or runny nose	146.00	31.00	45.00	15.00	13.00	1.87	1.00	1.21
Smell disorders (hyposmia or parosmia)	169.00	29.00	25.00	12.00	15.00	1.70	1.00	1.19
Swallowing or taste dysfunctions	165.00	41.00	15.00	16.00	13.00	1.68	1.00	1.16
Tinnitus, ear pain, dysphonia and sore throat	168.00	38.00	30.00	7.00	7.00	1.59	1.00	1.00
Skin rash	215.00	18.00	12.00	4.00	1.00	1.23	1.00	0.65
Sleep disorders	171.00	32.00	23.00	12.00	12.00	1.65	1.00	1.13
Mood disorders (general malaise)	140.00	41.00	40.00	15.00	14.00	1.89	1.00	1.21
Long cold	151.00	37.00	37.00	14.00	11.00	1.79	1.00	1.15
Depression or anxiety	160.00	41.00	25.00	12.00	12.00	1.70	1.00	1.13
Hair loss	191.00	30.00	12.00	9.00	8.00	1.45	1.00	0.97

Table S3. How much have the declared symptoms affected your daily life? (complete absolute frequencies).

1	2	3	4	5	mean	median	sd
70.00	71.00	69.00	28.00	12.00	2.36	2.00	1.14

Table S4. Have you experienced difficulties in resuming or performing the following activities? (complete absolute frequencies).

Activity	1	2	3	4	5	mean	median	sd
Work	107.00	68.00	49.00	17.00	9.00	2.01	2.00	1.11
Study	115.00	50.00	41.00	29.00	15.00	2.12	2.00	1.27
Household chores	120.00	61.00	46.00	17.00	6.00	1.91	2.00	1.07
Recreational activities	131.00	52.00	35.00	16.00	16.00	1.94	1.00	1.22
Mobilization	167.00	38.00	27.00	10.00	8.00	1.62	1.00	1.04
Social relationships	150.00	36.00	36.00	14.00	14.00	1.82	1.00	1.20
Health management	147.00	47.00	34.00	16.00	6.00	1.75	1.00	1.07
Self-care (such as washing, dressing)	188.00	33.00	18.00	8.00	3.00	1.42	1.00	0.85

Table S5. Today, do you believe that your life has changed following Covid-19 disease? (complete absolute frequencies).

1	2	3	4	5	mean	median	sd
96.00	69.00	44.00	21.00	20.00	2.20	2.00	1.26
